# DNA Methylation Near *DLGAP2* May Mediate the Relationship between Family History of Type 1 Diabetes and Type 1 Diabetes Risk

**DOI:** 10.1155/2023/5367637

**Published:** 2023-09-11

**Authors:** Randi K. Johnson, Amanda J. Ireton, Patrick M. Carry, Lauren A. Vanderlinden, Fran Dong, Alex Romero, David R. Johnson, Debashis Ghosh, Fan Yang, Brigitte Frohnert, Ivana V. Yang, Katerina Kechris, Marian Rewers, Jill M. Norris

**Affiliations:** ^1^Department of Biomedical Informatics, School of Medicine, University of Colorado Anschutz Medical Campus, Aurora, CO, USA; ^2^Department of Epidemiology, Colorado School of Public Health, University of Colorado Anschutz Medical Campus, Aurora, CO, USA; ^3^Colorado Program for Musculoskeletal Research, Department of Orthopedics, University of Colorado Anschutz Medical Campus, Aurora, CO, USA; ^4^Barbara Davis Center for Diabetes, School of Medicine, University of Colorado Anschutz Medical Campus, Aurora, CO, USA; ^5^Department of Biostatistics and Informatics, Colorado School of Public Health, University of Colorado Anschutz Medical Campus, Aurora, CO, USA

## Abstract

Given the differential risk of type 1 diabetes (T1D) in offspring of affected fathers versus affected mothers and our observation that T1D cases have differential DNA methylation near the imprinted *DLGAP2* gene compared to controls, we examined whether methylation near *DLGAP2* mediates the association between T1D family history and T1D risk. In a nested case–control study of 87 T1D cases and 87 controls from the Diabetes Autoimmunity Study in the Young, we conducted causal mediation analyses at 12 *DLGAP2* region CpGs to decompose the effect of family history on T1D risk into indirect and direct effects. These effects were estimated from two regression models adjusted for the human leukocyte antigen DR3/4 genotype: a linear regression of family history on methylation (mediator model) and a logistic regression of family history and methylation on T1D (outcome model). For 8 of the 12 CpGs, we identified a significant interaction between T1D family history and methylation on T1D risk. Accounting for this interaction, we found that the increased risk of T1D for children with affected mothers compared to those with no family history was mediated through differences in methylation at two CpGs (cg27351978, cg00565786) in the *DLGAP2* region, as demonstrated by a significant pure natural indirect effect (odds ratio (OR) = 1.98, 95% confidence interval (CI): 1.06–3.71) and nonsignificant total natural direct effect (OR = 1.65, 95% CI: 0.16–16.62) (for cg00565786). In contrast, the increased risk of T1D for children with an affected father or sibling was not explained by DNA methylation changes at these CpGs. Results were similar for cg27351978 and robust in sensitivity analyses. Lastly, we found that DNA methylation in the *DLGAP2* region was associated (*P* < 0.05) with gene expression of nearby protein-coding genes *DLGAP*2, *ARHGEF10*, *ZNF596*, and *ERICH1*. Results indicate that the maternal protective effect conferred through exposure to T1D *in utero* may operate through changes to DNA methylation that have functional downstream consequences.

## 1. Introduction

Type 1 diabetes (T1D) is a chronic autoimmune disease that affects the individual throughout their lifetime, contributing to a large healthcare burden [1]. While both genetics and environment are involved in the etiology of the disease, their exact roles have not been elucidated. A family history of T1D is associated with an increased risk of developing T1D; however, the strength of this risk depends on the type of relative with T1D. Several studies, including our own Diabetes Autoimmunity Study in the Young (DAISY), have found a higher risk of T1D in the children of affected fathers compared with the children of affected mothers [2–7]. Given that parents are equally likely to transmit diabetes susceptibility alleles and thus should also transmit T1D risk equally, this family history association pattern is suggestive of a parent-of-origin effect (POE) whereby maternal T1D confers some protection from T1D.

POE refers to a class of genetic effects where phenotypic expression in the offspring is dependent on whether the transmission originated from the mother or father. POE can arise due to several mechanisms [8], including differential gene expression due to genomic imprinting via methylation, the influence of the maternal in utero environment on fetal development, and variation in the maternally inherited mitochondrial genome. The role of genomic imprinting processes in T1D risk remains controversial, with evidence that both supports [9] and opposes [10, 11] the hypothesis. Recent population-based evidence suggests the maternal environment significantly contributes to T1D phenotypic variability, but genomic imprinting does not [12]. However, none of the previous studies examining the role of a maternal protective effect in T1D have used epigenetic data, the mechanism by which some POEs operate.

We had the unique opportunity to explore this intriguing hypothesis in a highly targeted way using findings from an epigenome-wide association study (EWAS) of T1D in DAISY [13]. We identified several regions located near the maternally imprinted gene *DLGAP2* [14–17], where longitudinal DNA methylation differed between T1D cases and controls. Based on this observation, we hypothesized that the association between family history of T1D and risk of T1D in the offspring that was observed in DAISY [7] could be explained by (i.e., is mediated by) differential DNA methylation in this region. We also examined the functional impact of differential DNA methylation near the *DLGAP2* region with gene expression using RNA-seq data available on a subset of DAISY participants.

## 2. Materials and Methods

### 2.1. Study Design and Population

The prospective DAISY cohort follows 2,547 Colorado children who are at high risk for developing T1D. Beginning in 1993, participants were identified and recruited from population-based newborn screening at St. Joseph's Hospital in Denver, CO, USA and from unaffected first-degree relatives of T1D patients. Of 31,881 screened newborns, children carrying human leukocyte antigen (HLA) haplogenotypes DR3/4, DQB1 ^*∗*^ 0302, DR3/3, and DR4/4, DQB1 ^*∗*^ 0302 and a sample of those with DR4/DRx, DQB1 ^*∗*^ 0302, or DR3/DRx (where DRx ≠ DR3 or DR4) were invited to participate in DAISY. The HLA-DR3/4 haplogenotype confers the highest risk for T1D. Participants are followed to islet autoimmunity (IA) and T1D. IA is defined as persistent autoantibodies to at least one of four antigens (insulin, GAD, IA2, and ZnT8) [18]. In order to determine IA, clinic visits and blood collection are conducted at ages 9, 15, and 24 months and annually thereafter. If a child tests positive for an autoantibody, their clinic visit frequency increases to every 3–6 months. The follow-up for the participant ends when they are diagnosed with Stage 3 T1D by a physician using the standard definition [19]. Additional details of screening and recruitment are available elsewhere [20, 21].

DAISY conducted a nested case–control study of 87 T1D cases and 87 controls to investigate high-dimensional genomics markers (e.g., metabolomics, DNA methylation). Controls, who were not positive for IA or T1D at the time of IA seroconversion of the case, were frequency matched to the cases on age at IA seroconversion, race–ethnicity and sample availability. We also generated RNAseq data for 138 DAISY children with samples collected pre- and post-seroconversion as described in detail in Carry et al. [22], including 55 children who were also selected for the methylation study. Race–ethnicity was categorized into non-Hispanic White and other, as there were no minority groups large enough to examine separately. The DAISY protocol complies with relevant ethical guidelines, including informed consent and assent of participants, and was approved by the Colorado Multiple Institutional Review Board (COMIRB 92-080).

### 2.2. Family History of T1D

Subjects were categorized into three family history groups based on the T1D status of first-degree relatives: (1) mother with T1D, (2) father or sibling with T1D, and (3) no first-degree relative with T1D. We previously showed in DAISY that participants with affected siblings have a comparable risk for T1D as participants with an affected father [7]; therefore, these two groups were combined to improve statistical power. Subjects who had an affected mother and father (*n* = 1) or an affected mother and a sibling (*n* = 5) were grouped into the mother category. Ten subjects had both an affected father and sibling. In all situations in which the DAISY child had an affected mother, the mother had T1D when she was pregnant with the child.

### 2.3. Selection of Candidate Mediators (CpGs)

An epigenome-wide screen of DNA methylation at multiple time points (up to five) prior to T1D diagnosis was conducted in the cases and controls using either the Illumina 450 K or the EPIC chip as described previously [13]. Data preprocessing and quality control were conducted in parallel on both platforms, including filtering to exclude CpGs on sex chromosomes, with SNPs in the CpG, or with low methylation range, which has poor reproducibility in epidemiological studies. CpGs were annotated to the nearest gene on build hg19 from Ensembl. The full processing pipeline is described in Vanderlinden et al. [23]. Two differentially methylated positions (DMP) and 28 differentially methylated regions (DMR) between T1D cases and controls were identified from this EWAS [13]. One of the DMPs and four of the DMRs were located in a region on chromosome eight that contained a number of genes (i.e., *ARHGEF10*, *RP11-43A14.1*, *RP5-855D21.1*, *ZNF596*, *ERICH1*, *RP11-439C15.4*), including *DLGAP2*, a gene that is known to be maternally imprinted [14]. We will refer to this region as the *DLGAP2* region. We selected the CpG site of the DMP and the 11 CpG sites within the four DMRs for these analyses (Table S1).

To obtain a nonimprinted region comparison, we selected a DMR (containing *HOPX*) from Johnson et al. [13] with similar significance and strength of association as those in the *DLGAP2* region, but that was not known to be imprinted [24]. We examined the five CpGs within the *HOPX* DMR (Table S1).

### 2.4. Gene Expression Profiling and Data Processing

For gene expression profiling, poly-A selected RNA is isolated from blood collected in Tempus tubes. The RNA was processed by the University of Colorado Genomics and Microarray Core facility, and paired-end sequence reads were generated using the Illumina NovaSEQ 600™ system. Data processing is described in detail in Carry et al. [22] and includes adapter trimming, alignment, transcript quantification, removal of unwanted (technical) variation, normalization, and transformation. The final log_2_ transformed data were used in all statistical analyses.

### 2.5. Statistical Analysis

**Figure 1:**
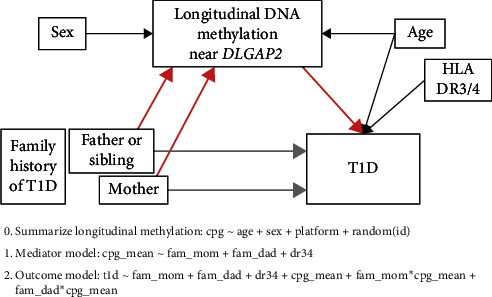
Mediation conceptual model and analytical approach. The directed acyclic graph shows the hypothesized relationships between family history of T1D (exposure), DNA methylation (mediator), and risk of T1D (outcome). Colors of the arrows represent the indirect (pink) and direct (tan) mediation effects. We implemented three models to first summarize longitudinal DNA methylation over time and then combined results from the mediator and outcome models in a counterfactual framework to estimate the causal indirect and direct effects. To accommodate possible interaction between the family history of T1D and DNA methylation, the outcome model includes an interaction term, and indirect and direct effects are reported separately for subjects with affected mothers and subjects with affected fathers or siblings, both compared to subjects with no family history of T1D.

Mediation analysis seeks to establish the pathway linking an exposure to an outcome by partitioning the total effect of an exposure on the outcome into the effect of the exposure that acts through a set of intermediate variables (indirect effect) and the effect of the exposure that is unexplained by those mediators (direct effect).  Figure 1 provides an overview of the conceptual mediation model and approach. For each CpG within the *DLGAP2* and *HOPX* regions, we fit three models. Based on prior knowledge, age may be a confounder of the mediator–outcome relationship. Sex is associated with methylation and was included as a precision variable. The high-risk HLA DR3/4 genotype explains much of the variation in T1D risk and was included as a precision variable.

First, the longitudinal aspect of the methylation data was reduced to one DNA methylation value per subject by taking the subject-specific random intercept from a mixed effects linear model predicting DNAm *M*-values as a function of time (age) as a random effect and platform (450 K vs. EPIC) and sex as fixed effects. These subject-specific intercepts represented the mean DNA methylation over time (age) and were used in the subsequent two models [25, 26]: (1) the mediator model, a weighted linear regression using robust standard errors for the effect of family history of T1D on DNA methylation adjusting for high-risk DR3/4 genotype, and (2) the outcome model, logistic regression for the effect family history of T1D on T1D risk, including terms for the interaction between family history of T1D and DNA methylation, DNA methylation, and high-risk DR3/4 genotype. The incidence of T1D in DAISY is approximately 6%, which was used to appropriately account for the balanced case–control design in the mediator model by down-weighting cases (0.06/0.50 = 0.12) and up-weighting controls ((1–0.06)/(1–0.50) = 1.88) [25, 26].

Estimates from the mediator and outcome model were combined using the counterfactual framework [25, 26] to estimate the effect of T1D family history on T1D risk that acts through DNA methylation (pure natural indirect effect, PNIE) and the effect of T1D family history on T1D risk that is unexplained by DNA methylation (total natural direct effect, TNDE). Given the potential for exposure–mediator interaction, PNIE, and TNDE are reported separately by category of T1D family history, comparing those with a mother with T1D and those with a father or sibling with T1D to subjects with no family history of T1D.

These mediation analyses assume no unmeasured confounding of the exposure–outcome, mediator–outcome, and exposure–mediator relationships and that none of the mediator–outcome confounders are affected by the exposure [26]. We conducted a sensitivity analysis to assess the robustness of our results to unmeasured confounding by calculating the mediational *E*-value for indirect and direct effects [27]. The *E*-value is the minimum strength of association an unmeasured confounder would need to have with the mediator and outcome in order to explain away the observed effects. Mediation also assumes temporal relationships between the exposure, mediator, and outcome, an assumption that is met by the nature of the prospective study design.

Finally, to understand whether DNA methylation has a functional impact on downstream gene expression and to provide orthogonal evidence on the robustness of mediation results, we performed expression quantitative trait methylation (eQTM) analysis among 55 DAISY children with both DNA methylation and RNAseq data available from a single study visit. All samples were obtained following the onset of IA. Multiple variable linear regression models were used to test the association between methylation levels at each CpG and log_2_ gene expression values. To capture the total effect of methylation across CpG sites within a region, we used a principal component analysis to identify a single variable, the first PC, that captured the most variability in methylation values across the entire DMR. We then tested the association between the 1st PC (of the DMR) and all genes with a transcription start site (TSS) that was ±1 Mb from the midpoint of the DMR. Age and sex were adjusted for in all linear regression models.

Analyses were conducted using R version 4.2.1. Given the targeted nature of these hypotheses (testing mediation and eQTM for probes within four DMRs near DLGAP2 and one DMR in HOPX), we did not adjust for multiple comparisons. Significance was evaluated at nominal *P* < 0.05 for main effects and at *P* < 0.1 for interaction terms.

## 3. Results

The mean age at T1D diagnosis in the cases was 9.7 years (Table 1). Compared to controls, a greater proportion of T1D cases had the high-risk HLA-DR3/4 genotype (48.3% vs. 19.5%) and had a father or sibling with T1D (56.3% vs. 35.6%). Sex and NHW ethnicity were similarly distributed between cases and controls. From multivariable logistic regression of the effect of family history on T1D risk, the overall risk for T1D (the total effect) is higher for subjects with affected fathers or siblings (OR = 3.36, 95% CI: 1.58–7.14) than for subjects with affected mothers (OR = 1.79, 95% CI: 0.64–5.00), both compared to those with no a family history of T1D.

From the mediator model, family history of T1D was associated with DNA methylation at *P* < 0.05 for eight probes in the *DLGAP2* region (Table 2), including cg02946697 and cg25674613 near *CTD-2281E23.1*; cg00565786 and cg27509052 near *CTD-2281E23.2*; cg19309499 near *CTD-2281E23.3*; and cg11192059, cg22763586, and cg27351978 near *DLGAP2*. Offspring of mothers with T1D had significantly increased methylation levels at each of these eight probes compared to children with no family history of T1D (all 95% CIs > 0). However, there was no difference in methylation between children with affected fathers or siblings and children with no family history (all 95% CIs cross 0). The other four probes in the *DLGAP2* region showed similar, though nonsignificant, trends of associations. No probes within the *HOPX* nonimprinted control region had significant associations with a family history of T1D (all *P* > 0.05).

From the outcome model, we identified nine probes in the *DLGAP2* region where there was a differential effect (interaction *P* < 0.1) of methylation (mediator) on T1D risk (outcome) by family history of T1D (exposure). To better visualize this exposure–mediator interaction, we rearranged the methylation and T1D terms to rerun the full outcome model as a linear regression and present the predicted methylation values by family history and T1D in Figure S1. In DAISY participants with a mother with T1D, there was decreased DNA methylation at each CpG site for T1D cases compared to controls. However, for those with a father or sibling with T1D or with no T1D relative, there was an opposite relationship between DNA methylation and T1D case status—cases had increased methylation or no difference in methylation compared to controls. This pattern of association was the same for all nine CpGs with significant mediator–exposure interaction terms, including cg02946697, cg08285446, cg24513387, and cg25674613 near *CTD-2281E23.1*; cg00565786 and cg27509052 near *CTD-2281E23.2*; and cg11192059, cg22763586, and cg27351978 near *DLGAP2*. No probes within the *HOPX* nonimprinted control region showed evidence of differential effects of methylation on T1D risk by family history of T1D (all exposure ^*∗*^ mediation interaction *P* > 0.1).

Parameter estimates from the mediator and outcome model (including exposure–mediator interaction) were combined to estimate the effect of T1D family history on T1D risk that acts through DNA methylation (PNIE), and the effect of T1D family history on T1D risk that is unexplained by DNA methylation (TNDE), as shown in Table 3. We found that the increased risk of T1D for subjects with affected mothers compared to those with no family history of T1D was mediated through changes to DNA methylation at cg00565786, as demonstrated by a significant PNIE (OR = 1.98, 95% CI: 1.06–3.71,) and nonsignificant TNDE (OR = 1.65, 95% CI: 0.16–16.62). In contrast, the increased risk of T1D for subjects with an affected father or sibling was not explained by DNA methylation changes, as demonstrated by a nonsignificant PNIE (OR = 1.22, 95% CI: 0.84–1.78) and a significant TNDE (OR = 2.84, 95% CI: 1.16–6.99). Most of the other 11 CpG sites in the *DLGAP2* region showed similar trends for PNIE and TNDE in causal mediation analyses, though only one additional probe (cg27351978) reached statistical significance for the maternal PNIE. The directions of indirect and direct effects (and statistical significance) were similar, whether estimated with or without the additional contribution of the high-risk HLA DR3/4 covariate.

These effects appear robust in sensitivity analyses. First, we performed meditational *E*-value analyses—to completely explain away the observed maternal indirect effects, an unmeasured confounder would need to be associated with both methylation and T1D with an association equal or higher to 3.38 (for cg00565786) and 4.16 (for cg27351978). No *HOPX* probes showed evidence of mediation. Second, we examined the impact of combining children with an affected sibling (*N* = 38) into the same category as those with an affected father (*N* = 42). We accomplished this by re-performing causal mediation analyses: (1) to exclude those with an affected sibling from the analysis and (2) to consider those with affected siblings in their own group. For probes near DLGAP2, indirect and direct effects were similar, whether combining those with affected siblings and father or excluding the siblings, though with expected changes to power due to decreased sample size (Figure S3). Similarly, mediation effects estimated separately among children with an affected sibling most resembled the effects for those with affected fathers. Results of sensitivity analyses indicate that the maternal effect conferred through exposure to T1D *in utero* may operate through changes to DNA methylation near *DLGAP2*.

Lastly, we examined the functional impact of differential DNA methylation near the *DLGAP2* region with gene expression. Participant characteristics of the RNAseq substudy were similar to the full study population (Table S2). There were 26 genes where the TSS was located within 1 Mb of the CpG or midpoint of the DMR, which were tested in eQTM analyses. Of these, we identified seven unique genes that were significantly correlated (i.e., significant eQTMs with nominal *P* < 0.05) with one or more methylation CpGs or the DMR (Table 4). The locations of these genes on chromosome 8 are shown in Figure S2.

Both CpGs that mediated the effect of exposure to maternal T1D on offspring T1D risk were associated with gene expression. Increased DNA methylation at cg00565786 was associated with increased expression of the protein-coding genes *ARHGEF10* and *ZNF596* and the antisense *RP5-855D21.1*. Increased DNA methylation at cg27351978 was associated with decreased expression of the sense intronic *RP11-43A14.1*. The association between cg02946697, an open sea CpG near gene *RP11-43A14.1*, was the strongest methylation-gene expression effect. Two CpGs have SNPs that are close by (i.e., rs58756222 near cg02946697 and rs80034362 near cg08285446); DNA methylation in these sites may be under genetic control (i.e., methylation quantitative trait loci). These results indicate that changes to DNA methylation in the *DLGAP2* region correspond to changes in gene expression in the region, including at *ARHGEF10*, *RP11-43A14.1*, *RP5-855D21.1*, *ZNF596*, *ERICH1*, *RP11-439C15.4*, and *DLGAP2*.

## 4. Discussion

Prior studies have established that exposure to maternal T1D confers lower T1D risk to the offspring than exposure to paternal T1D [2–7] and that T1D is preceded by differences in DNA methylation near the imprinted *DLGAP2* gene [13]. We hypothesized that DNA methylation served as a biological link between this maternal protective effect and offspring T1D risk. By conducting a causal mediation analysis, we identified significant indirect effects indicating that the risk conferred through exposure to maternal T1D may operate through changes to DNA methylation near *DLGAP2*. In contrast, only significant direct effects were identified for those with affected fathers, indicating that the increased risk conferred by exposure to paternal T1D operates through mechanisms other than DNA methylation near these regions.

We showed that DNA methylation near *DLGAP2* mediates the maternal POE in T1D risk; however, we are unable to distinguish whether these effects are genetic or environmental in origin. In all situations in which the DAISY child had an affected mother, the mother had diabetes when she was pregnant with the child, thus exposing the child to a diabetic *in utero* environment. DNA methylation may mark the silencing of a gene based on the parent of origin (e.g., genomic imprinting, a process that occurs during fetal development [8]), or it may reflect exposure to the diabetic in utero environment [28]. None of the *DLGAP2* region probes were found to be under genetic control as either cis- or trans-quantitative trait loci in whole blood [24] or human pancreatic islets [29]. We do not have parental genotypes nor parental methylation in this region and are therefore unable to discern what the child inherited from which parent. However, maternal environmental effects reportedly are stronger than maternal genetic effects in the risk of T1D in the offspring [12]. Many common maternal exposures have well-established impacts on DNA methylation, including nutrition [30, 31], glucose control [32], and smoking during pregnancy [33, 34]. Furthermore, *DLGAP2* DNA methylation can reflect environmental exposures such as cannabis use [35] and alcohol dependence [36]. While birthweight is often used as a proxy for exposure to poor intrauterine environment in pregnancy, the measure is less informative in T1D. Babies born to mothers with T1D have larger sizes for gestational age and are more likely to be delivered via C-section than babies born to non-T1D mothers [37]. Birthweight was not associated with T1D risk in DAISY, suggesting this will not explain the effects described herein [7]. Future studies should seek to disentangle the upstream genetic versus environmental influences on this POE.

Methylation at the DLGAP2 locus likely has downstream functional consequences. DNA methylation at a *DLGAP2* locus has been shown to affect *DLGAP2* gene expression in vitro [36] and in DAISY subjects (Table 4). *DLGAP2* encodes the SAP90/PSD-95-associated protein 2 (SAPAP2) that is involved in neuronal synaptic function and has been associated with autism spectrum disorder [38], schizophrenia [39], and Alzheimer's disease [40]. While *DLGAP2* is not a traditional diabetes susceptibility locus, growing evidence suggests its expression may play a role in diabetogenesis. In addition to our own prior report of differential DNA methylation at this locus associated with T1D risk [13], placental DNA methylation at this locus has been causally linked to maternal insulin sensitivity during pregnancy [41]. *DLGAP2* is most highly expressed in the brain and testis but is also fairly highly expressed in key endocrine organs, including pituitary and thyroid glands (Human Protein Atlas, https://proteinatlas.org) [42]. Moreover, Dlgap2 expression in mouse beta cells is regulated by LKB1 and AMPK, which control beta cell differentiation and glucose homeostasis [43]. These prior findings connecting *DLGAP2* methylation to gene expression and *DLGAP2* expression in T1D-related tissues with downstream signaling effects suggest a relationship between *DLGAP2* and T1D risk that should be further explored.

Our original EWAS finding suggested that *higher* methylation levels in the *DLGAP2* region were associated with increased risk of T1D overall [13], but the interaction analysis that we performed prior to our mediation analysis suggests a very different relationship in children of mothers with T1D (Figure S1). Such an exposure–mediator interaction has been previously described in epigenetic mediation studies [44] and indicates that exposure to maternal T1D must occur for DNA methylation near *DLGAP2* to affect T1D risk. Failing to account for this interaction and a small sample size of only seven case–control pairs may explain why recent longitudinal DNA methylation studies in T1D risk did not replicate findings from our original EWAS [45]. A subsequent investigation in cord blood found no epigenome-wide significant effects of DNA methylation on T1D risk but found and replicated concordant effect directions for three of the 28 original DMRs using Reduced representation bisulfite sequencing (RRBS) and pyrosequencing technology [46]. Only the two probes near *ERICH-AS1* were measured by RRBS, which selectively targets high-density CpG regions [47]. The remaining 15 probes in these analyses, including all probes near *CTD-2281E23.1*, *CTD-2281E23.2*, *DLGAP2*, and *HOPX*, were not measured by RRBS, which precludes our ability to seek replication in the DIPP study. Until such data are available from The Environmental Determinants of Diabetes in the Young, TEDDY, [48] or the Environmental Determinants of Islet Autoimmunity, ENDIA, [49] studies, our study population remains the largest collection of children at-risk for T1D with methylation, expression, and exposure data obtained prior to the development of T1D.

**Figure 2:**
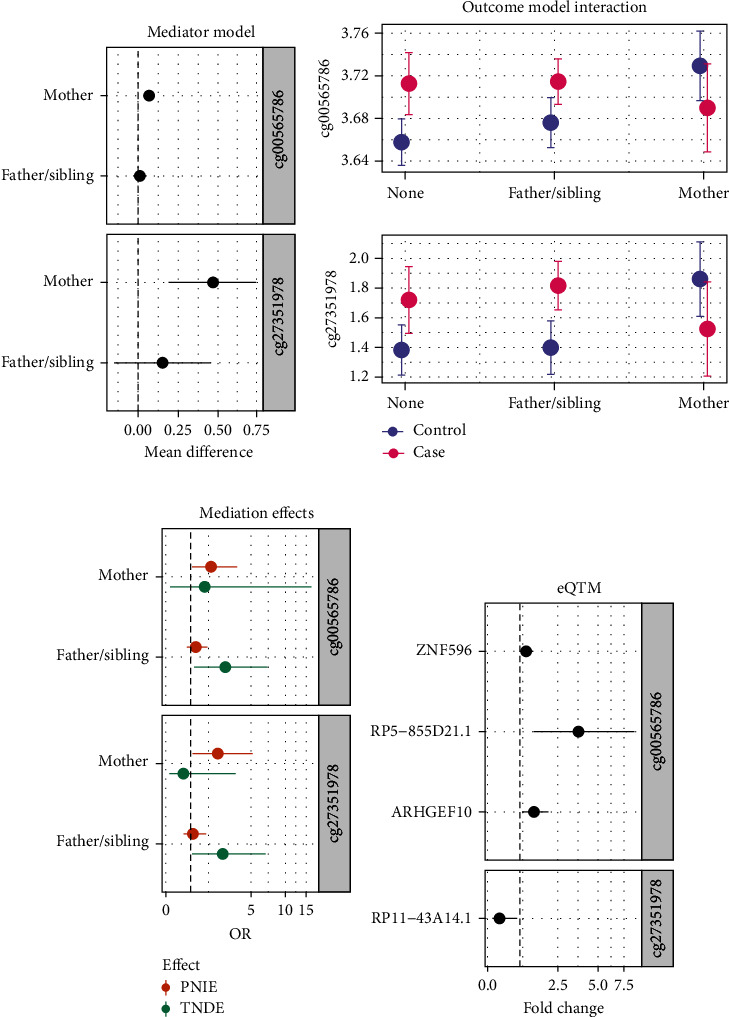
Analysis results for DLGAP2 probes with significant associations at each step (cg00565786, cg27351978). (a) Mediator model results from robust linear regression of the association between family history of T1D and methylation. (b) Outcome model interaction where the difference in methylation between T1D cases and controls differed by a family history of T1D—among those with an affected mother, cases had decreased methylation compared to controls, while the opposite relationship was seen among those with an affected father or no family history of T1D. (c) Mediation effect estimation combining the mediator and outcome model results. The significant indirect effect (PNIE) indicates that for those with an affected mother, the risk for T1D was mediated through DNA methylation at these locations. (d) Methylation associations with changes to cis-gene expression. PNIE, pure natural indirect effect; TNDE, total natural direct effect. All methylation was modeled on the *M*-value scale.

Prospective measurement of DNA methylation prior to T1D onset is a major strength for conducting causal mediation analyses—temporal relationships between exposure, mediator, and outcomes can be correctly inferred from the prospective follow-up. Cases and controls were well-matched on age at seroconversion, decreasing the possibility of any confounding due to age. We maximized our power by performing a very targeted analysis based on prior findings. Despite the small sample size, two of the 12 CpGs we tested, cg00565786 near *CTD-2281E23.2* and cg27351978 near *DLGAP2*, had significant results in each step of the mediation analysis (Figure 2). Due to the *a priori* targeted selection of probes, we assessed statistical significance at nominal levels; however, these two probes would pass a Bonferroni correction (at *α* = 0.05/5 DMRs). The patterns of effects we identified (e.g., the direction of effects) were remarkably similar across all probes tested in the *DLGAP2* region, and no associations were identified at any step of the mediation analysis for probes near the “control” nonimprinted *HOPX* region. Since our relatively small sample size may have prevented us from detecting small effects, and we may have failed to detect a methylation mediator that was not within the *a priori* selected imprinted and control region, larger studies should investigate methylation as mediators for POE in T1D across the epigenome.

## 5. Conclusions

Through causal mediation analyses in the prospective DAISY study, we demonstrated that the maternal protective effect conferred through exposure to T1D *in utero* may operate through changes to DNA methylation near *DLGAP2* that have functional downstream consequences. While further work is needed to replicate these findings and elucidate the upstream causes of this POE—due to genomic imprinting or *in utero* environmental exposures, these results indicate *DLGAP2* may be a promising therapeutic epigenetic target for T1D prevention.

## Figures and Tables

**Table 1:** Characteristics of T1D cases and frequency-matched controls in the longitudinal DAISY nested case–control study.

Characteristic	Case (*N* = 87)	Control (*N* = 87)	*P*-value
Family history of T1D, *N* (%)			0.023
Affected father or sibling	49 (56.3)	31 (35.6)	
Affected mother	10 (11.5)	16 (18.4)	
No first-degree relative with T1D	28 (32.2)	40 (46.0)	
High risk HLA-DR3/4 genotype, *N* (%)	42 (48.3)	17 (19.5)	6.2E-5
Male, *N* (%)	43 (49.4)	52 (59.8)	0.171
Non-Hispanic White, *N* (%)	77 (88.5)	76 (87.4)	0.816
Age at T1D diagnosis, mean years (SD)	9.7 (4.6)	n.a.	

**Table 2:** Mediator model results of the association between family history of T1D and DNA methylation from robust linear regression adjusted for high-risk HLA DR3/4 genotype.

Probe	DMR/DMP	Near gene	Affected mother			Affected father or sibling			*P*-value
			Estimate	Lower 95% CI	Upper 95% CI	Estimate	Lower 95% CI	Upper 95% CI	
Imprinted region (*DLGAP2*)									
cg02946697	DMR13	*CTD-2281E23.1*	0.24	0.12	0.36	−0.02	−0.14	0.10	1.08E-6
cg08285446	DMR13	*CTD-2281E23.1*	0.13	0.00	0.25	−0.01	−0.10	0.08	0.059
cg24513387	DMR13	*CTD-2281E23.1*	0.20	0.02	0.37	0.04	−0.09	0.18	0.098
cg25674613	DMR13	*CTD-2281E23.1*	0.32	0.10	0.54	0.03	−0.16	0.22	0.009
cg00565786	DMR10	*CTD-2281E23.2*	0.07	0.04	0.10	0.02	−0.01	0.05	8.6E-5
cg27509052	DMR10	*CTD-2281E23.2*	0.31	0.08	0.55	0.05	−0.14	0.25	0.026
cg19309499	DMP	*CTD-2281E23.3*	0.17	0.03	0.30	−0.01	−0.11	0.10	0.022
cg11192059	DMR9	*DLGAP2*	0.12	0.06	0.18	0.01	−0.08	0.09	1.2E-4
cg22763586	DMR9	*DLGAP2*	0.20	0.03	0.38	0.01	−0.12	0.15	0.046
cg27351978	DMR9	*DLGAP2*	0.46	0.19	0.74	0.05	−0.19	0.29	0.003
cg16922753	DMR5	*ERICH1-AS1*	0.32	0.05	0.58	0.07	−0.09	0.23	0.062
cg19530281	DMR5	*ERICH1-AS1*	0.21	0.04	0.38	0.05	−0.07	0.16	0.051
Nonimprinted region (*HOPX*)									
cg00493422	DMR2	*HOPX*	0.02	−0.16	0.20	0.12	−0.02	0.25	0.166
cg04085076	DMR2	*HOPX*	0.02	−0.16	0.20	0.05	−0.09	0.19	0.767
cg06771126	DMR2	*HOPX*	0.01	−0.16	0.19	0.04	−0.10	0.19	0.809
cg16975863	DMR2	*HOPX*	0.03	−0.04	0.10	0.04	−0.01	0.09	0.215
cg25456368	DMR2	*HOPX*	0.08	−0.13	0.29	0.14	−0.04	0.32	0.304

DMR, differentially methylated region, DMR identifier Johnson et al. [13], *Sci Rep*. DMP, differentially methylated position.

**Table 3:** Decomposed direct and indirect causal mediation effects for the effect of family history of T1D (exposure) on T1D risk (outcome) mediated through DNA methylation (mediator).

CpG	DMR/DMP	Near gene	Affected mother						Affected father or sibling					
			PNIE			TNDE			PNIE			TNDE		
			OR	Lower 95% CI	Upper 95% CI	OR	Lower 95% CI	Upper 95% CI	OR	Lower 95% CI	Upper 95% CI	OR	Lower 95% CI	Upper 95% CI
Imprinted region (*DLGAP2*)														
cg02946697	DMR13	*CTD-2281E23.1*	1.50	0.89	2.56	5.18	0.12	223.14	0.97	0.79	1.19	3.76	1.59	8.89
cg08285446	DMR13	*CTD-2281E23.1*	1.35	0.86	2.10	1.30	0.35	4.91	0.98	0.80	1.20	3.52	1.55	8.01
cg24513387	DMR13	*CTD-2281E23.1*	1.25	0.85	1.85	1.72	0.35	8.60	1.05	0.88	1.25	3.21	1.44	7.17
cg25674613	DMR13	*CTD-2281E23.1*	1.65	0.94	2.88	1.07	0.27	4.19	1.05	0.78	1.40	3.08	1.31	7.21
**cg00565786**	**DMR10**	***CTD-2281E23.2***	**1.98**	**1.06**	**3.71**	**1.65**	**0.16**	**16.62**	**1.22**	**0.84**	**1.78**	**2.84**	**1.16**	**6.99**
cg27509052	DMR10	*CTD-2281E23.2*	1.92	0.99	3.73	0.90	0.20	4.08	1.12	0.74	1.70	2.77	1.12	6.83
cg19309499	DMP	*CTD-2281E23.3*	1.59	0.95	2.67	1.12	0.34	3.70	0.98	0.73	1.31	3.87	1.59	9.39
cg11192059	DMR9	*DLGAP2*	1.63	0.95	2.79	2.49	0.16	39.10	1.02	0.74	1.42	2.97	1.30	6.78
cg22763586	DMR9	*DLGAP2*	1.76	0.93	3.32	0.99	0.24	4.19	1.04	0.72	1.51	3.13	1.33	7.37
**cg27351978**	**DMR9**	***DLGAP2***	**2.37**	**1.08**	**5.17**	**0.69**	**0.13**	**3.59**	**1.10**	**0.70**	**1.72**	**2.67**	**1.06**	**6.73**
cg16922753	DMR5	*ERICH1-AS1*	1.39	0.79	2.42	1.25	0.38	4.19	1.07	0.88	1.30	3.34	1.37	8.17
cg19530281	DMR5	*ERICH1-AS1*	1.78	0.89	3.56	0.92	0.24	3.58	1.13	0.81	1.59	3.01	1.17	7.76
Nonimprinted region (*HOPX*)														
cg00493422	DMR2	*HOPX*	1.05	0.70	1.58	1.89	0.61	5.85	1.31	0.91	1.89	2.77	1.18	6.48
cg04085076	DMR2	*HOPX*	1.04	0.72	1.51	2.12	0.57	7.87	1.11	0.82	1.49	3.29	1.41	7.65
cg06771126	DMR2	*HOPX*	1.02	0.74	1.41	2.19	0.58	8.26	1.08	0.83	1.43	3.33	1.46	7.61
cg16975863	DMR2	*HOPX*	1.29	0.73	2.28	1.36	0.41	4.57	1.40	0.91	2.17	2.45	0.96	6.24
cg25456368	DMR2	*HOPX*	1.13	0.82	1.54	2.02	0.54	7.60	1.22	0.90	1.66	2.90	1.27	6.64

PNIE (pure natural indirect effect) = the effect of T1D family history on T1D risk that acts through DNA methylation. TNDE (total natural direct effect) = the effect of T1D family history on T1D risk that is unexplained by DNA methylation. DMR, differentially methylated region, DMR identifier Johnson et al. [13], *Sci Rep*. DMP, differentially methylated position. Bold = probes displaying evidence of significant mediation through methylation for children of affected mothers (significant PNIE, nonsignificant TNDE) but not for children of affected fathers or siblings (nonsignificant PNIE, significant TNDE).

**Table 4:** Association between DNA methylation and nearby cis-gene expression in DAISY subjects.

Probe	Pos	Ensembl ID	Gene symbol	Gene start	Gene end	Strand	Biotype	Beta	*P*-value
Single probe analysis									
cg11192059	1650035	ENSG00000104728	*ARHGEF10*	1772142	1906807	1	Protein coding	1.49	0.019
cg27351978	1650172	ENSG00000254207	*RP11-43A14.1*	675188	675877	−1	Sense intronic	0.36	0.036
cg16922753	1113291	ENSG00000272240	*RP5-855D21.1*	184347	184887	−1	Antisense	0.3	0.010
cg00565786	1012465	ENSG00000104728	*ARHGEF10*	1772142	1906807	1	Protein coding	1.5	0.019
cg00565786	1012465	ENSG00000172748	*ZNF596*	182137	197342	1	Protein coding	1.21	0.050
cg00565786	1012465	ENSG00000272240	*RP5-855D21.1*	184347	184887	−1	Antisense	3.62	0.007
cg02946697	1273833	ENSG00000254207	*RP11-43A14.1*	675188	675877	−1	Sense intronic	0.24	0.002
cg08285446	1273856	ENSG00000104714	*ERICH1*	564746	688106	−1	Protein coding	0.92	0.032
cg24513387	1273604	ENSG00000254207	*RP11-43A14.1*	675188	675877	−1	Sense intronic	0.34	0.026
Regional analysis									
DMR PC1	1273706	ENSG00000198010	*DLGAP2*	1449532	1656642	1	Protein coding	1.47	0.023
DMR PC1	1273706	ENSG00000253764	*RP11-439C15.4*	1920736	1922803	−1	lincRNA	2.21	0.029

Pos, location of probe on chromosome 8, for the DMR, represents midpoint of the entire region. Biotype, gene type based on ensemble annotation. Beta, beta coefficient from the linear regression model, represents fold change in gene expression per 1 standard deviation increase in methylation *M*-value, values <1 represent negative association between methylation and gene expression, values >1 represent positive association between methylation and gene expression.

## Data Availability

The datasets generated during and/or analyzed during the current study are accessible through GEO Series accession number GSE142512.

## Supplementary Materials

Supplementary Materials Table S1: Selected *DLGAP2* and *HOPX* Probe Annotations. Table S2: Comparison of characteristics of the eQTM substudy compared to the full study population. Figure S1: Predicted DNA methylation at *DLGAP2* and *HOPX* probes from a linear model with interaction term allowing for different effects of methylation on type 1 diabetes by family history of type 1 diabetes. Figure S2: Genes (primary isoform) where expression is correlated with DNA methylation in the chromosome eight region near *DLGAP2* among DAISY subjects. Figure S3: Causal mediation results for *DLGAP2* probes from sensitivity analyses recategorizing children with a sibling with T1D.
