# Supplementary material for: DNA Methylation Near DLGAP2 May Mediate the Relationship between Family History of Type 1 Diabetes and Type 1 Diabetes Risk
Source: Pediatr Diabetes. Author manuscript; Available in PMC 2024 May 17. (PMC11100224; doi:10.1155/2023/5367637)

DNA methylation near *DLGAP2* may mediate the relationship between family history of type 1 diabetes and type 1 diabetes risk

Johnson et al.

SUPPLEMENTARY MATERIALS

Supplemental Table 1: Selected DLGAP2 and HOPX Probe Annotations. *All CpGs showed hypermethylation in T1D cases compared to controls in REF 13 Johnson et al (2020), Sci Rep.*

Probe	Chr	Position	DMR/DMP	Near Gene
**Imprinted Region (*DLGAP2*)**				
cg02946697	chr8	1273833	DMR13	*CTD-2281E23.1*
cg08285446	chr8	1273856	DMR13	*CTD-2281E23.1*
cg24513387	chr8	1273604	DMR13	*CTD-2281E23.1*
cg25674613	chr8	1273808	DMR13	*CTD-2281E23.1*
cg00565786	chr8	1012465	DMR10	*CTD-2281E23.2*
cg27509052	chr8	1012324	DMR10	*CTD-2281E23.2*
cg19309499	chr8	1150488	DMP	*CTD-2281E23.3*
cg11192059	chr8	1650035	DMR9	*DLGAP2*
cg22763586	chr8	1649868	DMR9	*DLGAP2*
cg27351978	chr8	1650172	DMR9	*DLGAP2*
cg16922753	chr8	1113291	DMR5	*ERICH1-AS1*
cg19530281	chr8	1113432	DMR5	*ERICH1-AS1*
**Non-Imprinted Region (*HOPX*)**				
cg00493422	chr4	57547872	DMR2	*HOPX*
cg04085076	chr4	57547579	DMR2	*HOPX*
cg06771126	chr4	57547699	DMR2	*HOPX*
cg16975863	chr4	57548093	DMR2	*HOPX*
cg25456368	chr4	57547347	DMR2	*HOPX*

DMR = differentially methylated region, DMR identifier from REF 13 Johnson et al (2020), *Sci Rep*
DMP = differentially methylated position

Supplemental Table 2: Comparison of characteristics of the eQTM substudy compared to the full study population.

	eQTM Population n=55		Full Population n=174	
	**Freq | Mean**	**% | Stdev**	**Freq | Mean**	**% | Stdev**
**Family History, n (%)**				
Mom	9	16.4%	26	14.9%
None	24	43.6%	70	40.2%
Sibling/Dad	22	40.0%	78	44.8%
**Type 1 Diabetes Case, n (%)**	23	41.8%	87	50.0%
**Non-Hispanic White, n (%)**	43	78.2%	153	87.9%
**Female Sex, n (%)**	28	50.9%	79	45.4%
**HLA DR 3/4 Genotype, n (%)**	22	40.0%	59	33.9%
**Age at IA, mean (stdev)**	8.8	4.9	9.1	6.7

Supplemental Figure 1: Predicted DNA methylation at DLGAP2 and HOPX probes from a linear model with interaction term allowing for different effects of methylation on type 1 diabetes by family history of type 1 diabetes.

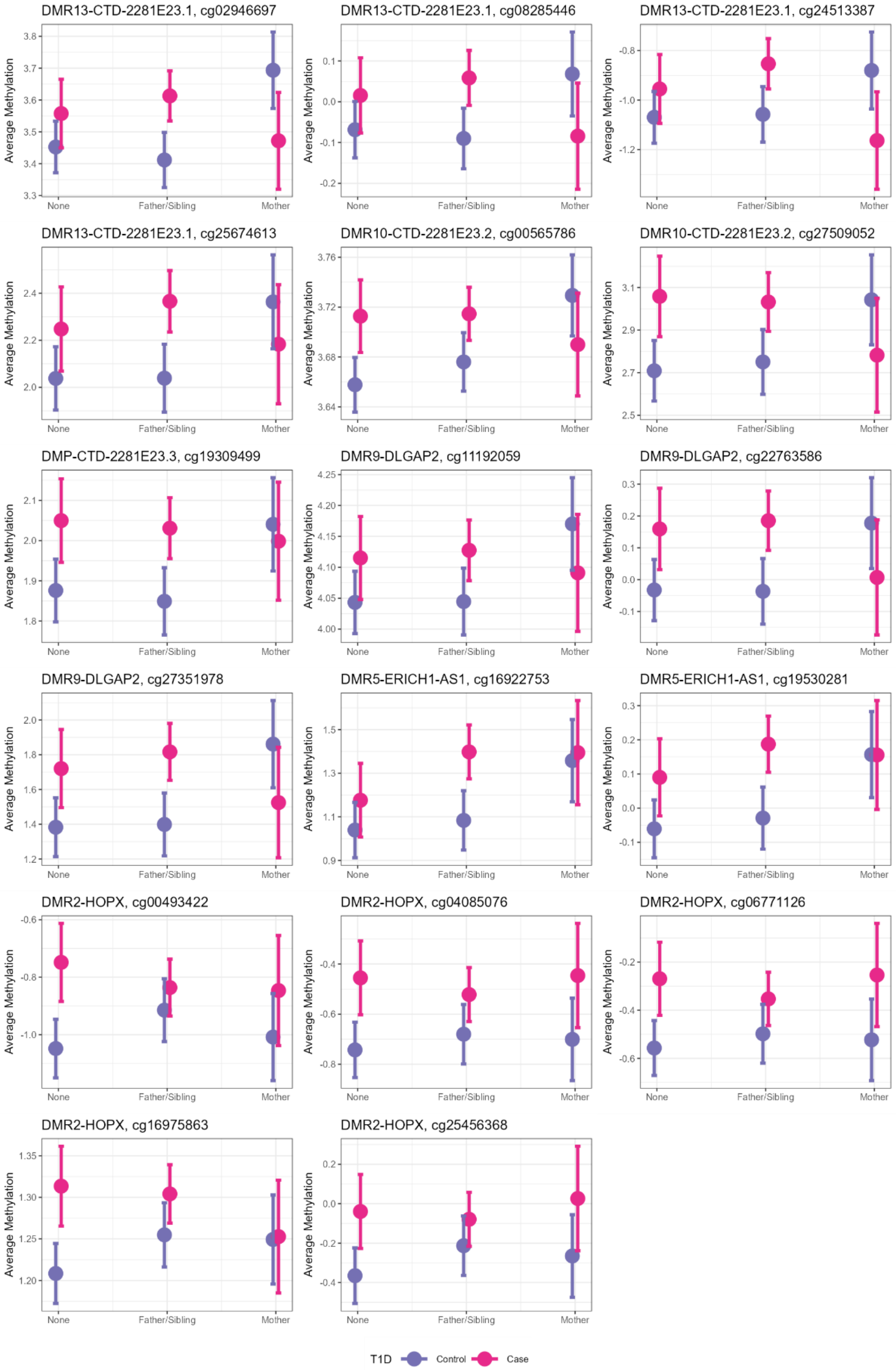

Supplemental Figure 2: Genes (primary isoform) where expression is correlated with DNA methylation in the chromosome 8 region near DLGAP2 among DAISY subjects. *Region on chromosome 8 identified in our previous epigenome-wide association study (EWAS) of type 1 diabetes in DAISY (REF 13). The top panel represents the position (kb) of the methylation region (black solid square) relative to the gene transcripts (green solid squares) identified in the secondary meQTM analysis. The bottom panel represents a more detailed visualization of the gene transcripts including exons (green). The x-axis represents the position (kb) of the genes within the region. There are multiple known isoforms for the genes; the figure displays the most biologically relevant or consensus transcript based on the Ensembl database. The red line on the ideogram, bottom of the figure, represents the location of this region on chromosome 8.*

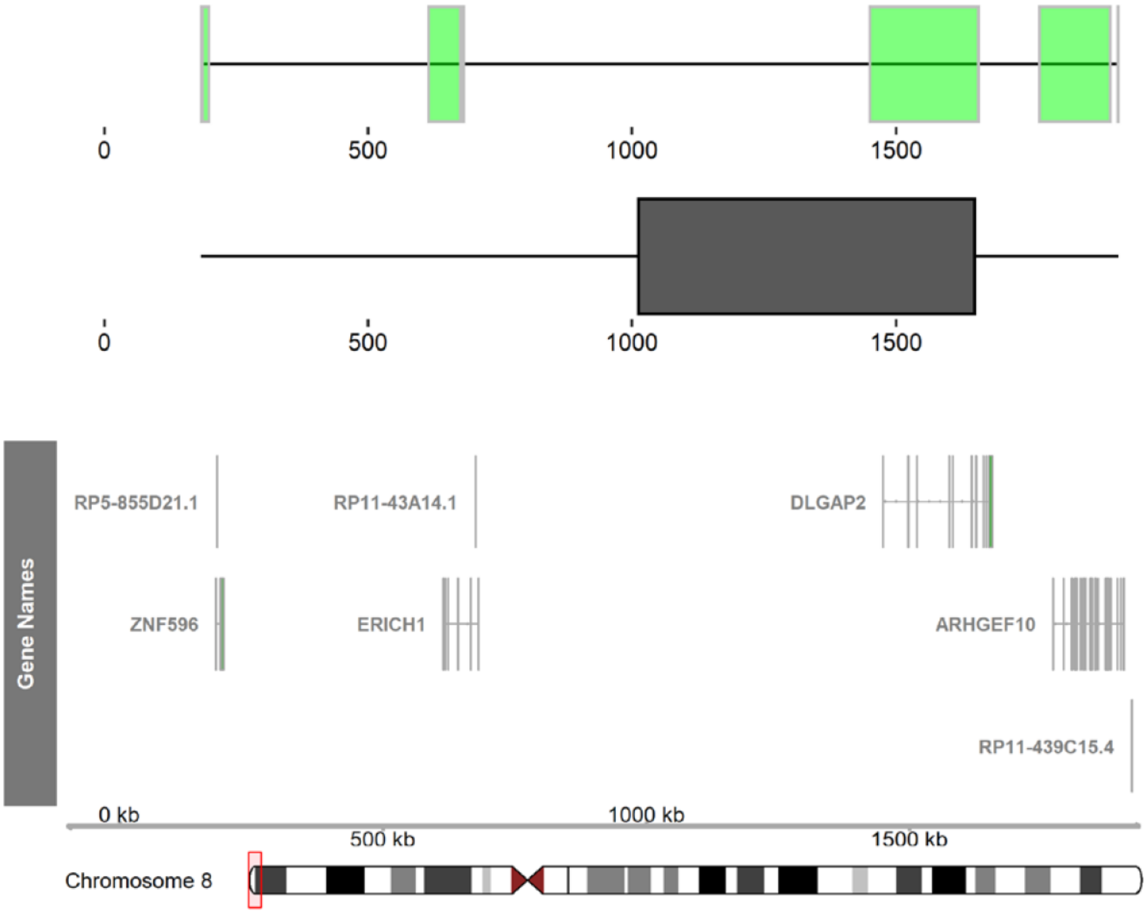

Supplemental Figure 3: Causal mediation results for DLGAP2 probes from sensitivity analyses recategorizing children with a sibling with T1D. *The "Combined" analysis is the main analysis, performed as described in the main text where those with a father or sibling with T1D were combined into one group. The same methodology was used for two sensitivity analyses, each incorporating a reclassification of those with affected siblings. In the "Excluded" analysis, children with a sibling with T1D (N=38) were excluded from analyses (new N=136). In the "Separated" analysis, the children with an affected sibling were treated as a separate group from those with affected fathers (N=42), mothers (N=26), or no family history (N=68). PNIE = pure natural indirect effect. TNDE = total natural direct effect.*

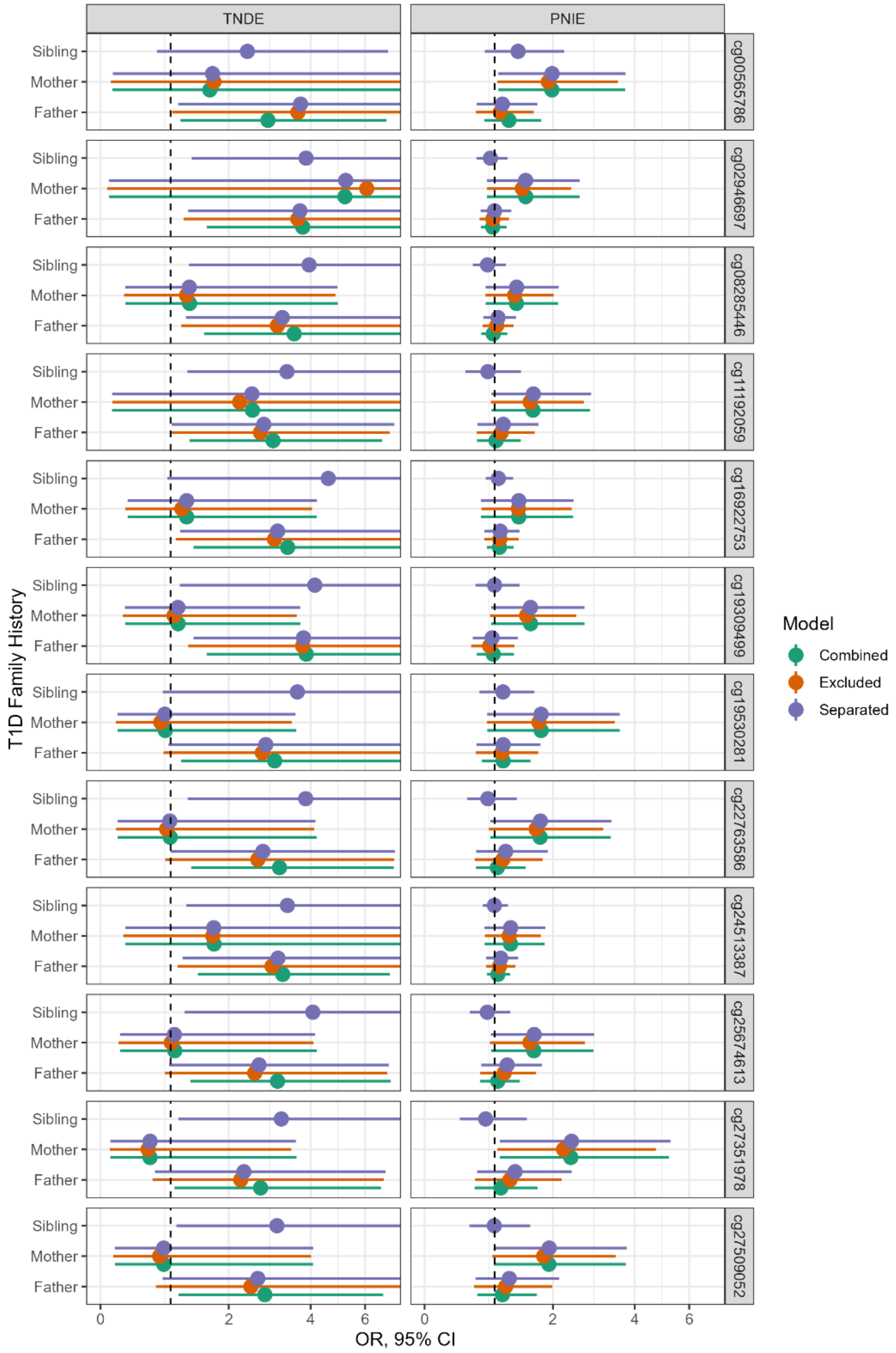

Supplement: Supplementary Figures and Tables [file NIHMS1990081-supplement-Supplementary_Figures_and_Tables.pdf]
